# Innovation of Surgical Techniques for Screw Fixation in Patients with Osteoporotic Spine

**DOI:** 10.3390/jcm11092577

**Published:** 2022-05-04

**Authors:** Haruo Kanno, Yoshito Onoda, Ko Hashimoto, Toshimi Aizawa, Hiroshi Ozawa

**Affiliations:** 1 Department of Orthopaedic Surgery, Tohoku Medical and Pharmaceutical University, Sendai 983-8536, Japan; hozawa@tohoku-mpu.ac.jp; 2 Department of Orthopaedic Surgery, Tohoku University School of Medicine, Sendai 980-8574, Japan; onodaworld@hotmail.com (Y.O.); hasshie@gmail.com (K.H.); toshi-7@ra2.so-net.ne.jp (T.A.)

**Keywords:** pedicle screw, percutaneous pedicle screw, hydroxyapatite granules, augmentation, osteoporosis, spine, screw loosening, spine surgery, minimally invasive spinal treatment, minimally invasive spine stabilization

## Abstract

Osteoporosis is a common disease in elderly populations and is a major public health problem worldwide. It is not uncommon for spine surgeons to perform spinal instrumented fusion surgeries for osteoporotic patients. However, in patients with severe osteoporosis, instrumented fusion may result in screw loosening, implant failure or nonunion because of a poor bone quality and decreased pedicle screw stability as well as increased graft subsidence risk. In addition, revision surgeries to correct failed instrumentation are becoming increasingly common in patients with osteoporosis. Therefore, techniques to enhance the fixation of pedicle screws are required in spinal surgeries for osteoporotic patients. To date, various instrumentation methods, such as a supplemental hook, sublaminar taping and sacral alar iliac screws, and modified screwing techniques have been available for reinforcing pedicle screw fixation. In addition, several materials, including polymethylmethacrylate and hydroxyapatite stick/granules, for insertion into prepared screw holes, can be used to enhance screw fixation. Many biomechanical tests support the effectiveness of these augmentation methods. We herein review the current therapeutic strategies for screw fixation and augmentation methods in the surgical treatment of patients with an osteoporotic spine.

## 1. Introduction

With an aging population and longer life span, osteoporosis is becoming increasingly common [1,2]. In patients with severe osteoporosis, instrumented fusion may result in screw loosening, implant failure or nonunion because of a poor bone quality and decreased pedicle screw stability as well as an increased graft subsidence risk [3,4,5]. Previous studies have shown that the rate of screw loosening ranges from <0.6% to 15% in non-osteoporotic patients and reaches 60% in osteoporotic subjects [6,7]. However, it is not uncommon for spine surgeons to perform instrumentation surgeries for osteoporotic spines. In addition, revision surgeries to correct failed instrumentation are becoming increasingly common in patients with osteoporosis. Thus, surgical techniques to reinforce pedicle screw fixation are required to obtain successful outcomes in spinal surgeries for osteoporotic patients.

To date, various options of surgical instruments, such as a spinal hook [8,9,10], sublaminar tape/band [11,12] and sacral alar iliac (S2AI) screws [13,14], and modified screwing techniques [15,16,17,18] have been made available for augmenting pedicle screw fixation. In addition, several materials, including polymethylmethacrylate (PMMA) [19,20] and hydroxyapatite (HA) stick/granules [21,22,23], for insertion into prepared pedicle holes, can be used to enhance screw fixation. Many clinical biomechanical tests support the effectiveness of these augmentation methods [8,9,10,11,16,20,22,23,24].

We herein review currently available therapeutic strategies for screw fixation and augmentation methods in the surgical treatment of patients with an osteoporotic spine.

## 2. Basic Principle of Pedicle Screw Fixation for Osteoporotic Spine

Appropriately sized and positioned pedicle screws are important for obtaining optimal fixation in the osteoporotic spine [25]. Larger diameter screws have been shown to improve the pullout strength of pedicle screws [3,26]. However, oversizing the pedicle should be avoided because some larger screws may result in pedicle fracture of the osteoporotic vertebrae [3,27]. It has also been reported that the length of the screw improves pullout strength [26]. In addition, undertapping the pedicle screw tract increases pullout strength [25,28,29]. The same diameter tapping of screws decreases the insertional torque and consequently reduces the pullout strength of the pedicle screw. Therefore, it has been recommended that the surgeon should undertap the path of the pedicle screw in patients with an osteoporotic spine [29].

Taken together, meticulous preoperative planning is essential for successful surgical outcomes. Surgeons should select the appropriate size of pedicle screws and carefully consider the number of screw placements and the number of fusion levels in order to obtain sufficient spinal stability and rigid screw fixation. Surgeons can use other surgical techniques to reinforce screw fixation, as described below (Table 1).

## 3. S2AI Screws

There is increasing evidence that pelvic fixation may become the standard method of various instrumented spine surgeries [30]. Pelvic fixation is an important method of ensuring stability at the base of long construct fusions and should be considered in patients with a long construct ending in the sacrum, those with associated risk factors for loss of distal fixation or a high risk of pseudarthrosis at L5–S1 and those undergoing three-column osteotomies or vertebral body resection in the lower lumbar spine [13,30]. In addition, pelvic fixation can also be indicated for patients with high-grade spondylolisthesis, unstable sacral fractures, sacral tumors and insufficiency fractures [13,30]. In corrective surgery for osteoporotic patients with severe spinal deformity in particular, pelvic fixation is a very useful method of obtaining rigid fixation [31].

Long constructs extending from the thoracic spine to the distal lumbar spine and/or sacrum result in large lever arms and cantilever forces, causing substantial stress at the base of the construct. Furthermore, pedicle screws placed at S1 have a significant risk of screw loosening, especially in elderly patients with osteoporosis, as the S1 pedicles in the sacrum largely include cancellous bone [13,24]. Therefore, pelvic fixation is very important for ensuring a stable construct base and maintaining surgical correction of the spinal deformity until bony fusion is achieved [13].

Although pelvic fixation can be performed using various surgical procedures, the currently available iliac screw fixation and S2AI screw fixation techniques are considered the most common methods. A previous study suggested that S2AI screw fixation in adults may have a lower risk of mechanical failure and complication than iliac screw fixation [30]. Indeed, S2AI screws inserted from the S2 alar crossing the sacroiliac joint into the ilium are considered to provide stronger fixation than iliac screws [14].

## 4. Intra-Sacral Buttress Screws

While pelvic fixation using S2AI screws provides strong stability at the base of the construct [14], the impact of S2AI screws on the sacroiliac joint remains uncertain in the long term. Recently, Fukuda et al., reported a novel fixation method using intra-sacral buttress screws (ISBSs) that is strongly stabilized by the sacral subchondral bone without penetration of the sacroiliac joint [32]. In this fixation method, a screw with a polyaxial head is inserted into the lateral sacral mass and assembled to the rod connected cephalad to the pedicle screws. The dorsal side of the screw can then be stabilized by the sacral subchondral bone at the sacroiliac joint with iliac buttress coverage, while the tip of the screw is anchored by the sacral cortex [32].

The connection of an intra-sacral rod to pedicle screws placed at the lumbar spine is a technique for lumbosacral fixation that was reported by Jackson and McManus [33]. ISBSs can be used as alternatives for the intra-sacral rod. The use of this alternative technique involving screws with polyaxial heads enables easier assembly of the rod and screws than with previous techniques [32]. ISBSs combined with conventional S1 pedicle screws are considered to provide a greater fixation strength at the base of long construct fusions than with S1 pedicle screws alone.

## 5. Cortical Bone Trajectory Screws

The cortical bone trajectory (CBT) screw technique is another pedicle screw insertion method described in 2009 by Santoni et al. [34]. The CBT screw fixation within the pedicle is targeted in a mediolateral path in the axial plane and a caudocranial path in the sagittal plane [34]. This screw trajectory engages the cortical bone and theoretically provides increased cortical bone contact, increased screw grip and a reduced reliance on trabecular bone [35]. CBT screws were reported to have a higher insertion torque than traditional pedicle screws in vivo [36]. Several biomechanical analyses have shown that the CBT screw/rod construct has favorable mechanical properties [37,38].

Traditional pedicle screw fixation requires extensive tissue dissection to expose the entry points and allow for lateral-to-medial screw trajectory insertion. In contrast, CBT screw insertion through a caudomedial starting point can reduce the length of incisions of the superior facet joints and paraspinal muscles [39,40]. Thus, CBT screw fixation can lead to posterior lumbar fusion surgery with reduced invasiveness [39,40].

## 6. Penetrating Endplate Screws

Previous studies have suggested that modified pedicle screw insertion techniques penetrating the superior endplate of the vertebral body can be useful for enhancing the screw fixation strength [15,16,17,41]. Recently, Matsukawa et al., reported the penetrating S-1 endplate screwing technique [15]. The penetrating S-1 endplate technique, through the medial entry point, is suitable for the connection of lumbar CBT screws. This trajectory engages with denser bone maximally by the screw penetrating the S-1 superior endplate through a more medial entry point than the traditional technique [15]. This screwing technique has favorable stability for lumbosacral fixation and reduces the potential risk of screw loosening and implant failure [15]. It also has several safety advantages, with the protrusion of the screw tip into the intervertebral disc space carrying no risk of causing neurovascular injury [15]. A biomechanical study demonstrated that the new technique demonstrated greater insertional torque than the traditional monocortical technique [15].

Minamide et al., compared fixation with an L-5 pedicle screw and an S-1 transdiscal screw connected by a rod to fixation with the standard L5–S1 construct [16]. They found that the transdiscal construct approach was stiffer than the standard pedicle screw fixation [16]. In addition, L5-S1 transdiscal screw fixation provided better functional and radiographic outcomes than conventional pedicle fixation for high-grade spondylolisthesis [42]. Furthermore, another study reported that transvertebral screws with purchase on two vertebral segments across multiple cortical layers can provide a high fusion rate in the thoracic spine [43].

Taken together, these findings suggest that the screw penetrating the endplate may be a useful procedure for maximizing the thread contact with the dense bone purchase and achieving solid spine fusion [41]. Indeed, it has been reported that penetrating endplate screws can be effective in posterior fusion surgery for patients with diffuse idiopathic skeletal hyperostosis (DISH) and spinal metastases [17].

## 7. Groove Entry Technique and Hooking Screw Technique

Previous studies suggested that the groove entry technique and hooking screw technique for thoracic percutaneous pedicle screw (PPS) fixation may provide greater screw stability than conventional pedicle screw fixation [18,44]. In these techniques, the PPS enters at a dorsal site craniolateral to the pedicle, where the cortical bone is thicker than at the caudal aspect of the pedicle, and passes obliquely through the thickened cortical bone [18]. These screwing techniques utilize the strength of posterior vertebral elements with purchase on the transverse and articular process and endplates, transfixing the pedicle in a diagonal fashion, and hugging the strong cortex of the neuroforamen and spinal canal [18]. The diagonal trajectory allows for longer screw insertion, and longer screws have more contact with bone and are better able to engage the anterior column than shorter ones [44]. Therefore, diagonal screw instrumentation, such as the groove entry technique and the hooking screw technique, may provide sufficient anchoring strength to prevent implant failure [18,44].

## 8. HA Stick and HA Granules

It has been reported that the placement of substances into the tapped screw hole increases the bone–metal interface friction force and enhances the mechanical strength of screw fixation [21,45,46,47,48]. Osteoconductive ceramic bone graft materials, such as HA and calcium phosphate, have received attention as clinically available biomaterials to increase the stability of screw fixation [45,49].

Several previous studies have suggested that an HA stick or HA granules inserted into the tapped screw hole may help enhance the initial strength of pedicle screw fixation [21,22,23]. For more than two decades, the augmentation technique of pedicle screws using an HA stick has been widely used in spinal surgeries in various countries [21,50]. Previous studies have indicated the biomechanical advantage and clinical usefulness of this augmentation technique [21,22,50].

Recently, we reported a new augmentation technique for PPS fixation using HA granules [23]. We developed a dedicated device for the percutaneous insertion of HA granules to perform this augmentation, using a guidewire with no additional skin incision [23]. The insertion device was approved as a medical device for surgical use, and we have applied such augmentation to PPS fixation for various surgeries in patients with an osteoporotic spine [22]. We have reported the biomechanical advantages of augmentation with HA granules for PPS fixation in a synthetic bone model [23]. In addition, our cadaveric analysis demonstrated that PPS fixation was significantly enhanced by augmentation with HA granules in osteoporotic lumbar spine [22]. Furthermore, PPS fixation augmented with HA granules can reduce the incidence of screw loosening and implant failure in patients with an osteoporotic spine [22,51].

## 9. PMMA

PMMA bone cements appear promising for the augmentation of pedicle screw fixation biomechanically in both osteoporosis and revision spine surgery models [19]. Burval et al., reported that pedicle screw augmentation with PMMA improves the initial fixation strength and fatigue strength of instrumentation in osteoporotic vertebrae [20]. Becker et al., demonstrated that the augmentation of fenestrated pedicle screws with PMMA provided higher pullout resistance than nonaugmented screws [52]. Chen et al. [53] demonstrated that prefilling PMMA with solid screws resulted in improved axial pullout strength compared with injecting PMMA through a fenestrated screw. A recent study reported a clinical usefulness of percutaneous pedicle screw fixation with cement augmentation in surgical treatment of thoracolumbar fractures in patients with ankylosing spondylitis [54].

However, there is a risk of cement extravasation leading to potentially neurological or cardiovascular complications with cement use [19,50]. In addition, cements, including PMMA, have exothermic properties that may induce bone necrosis and the degeneration of adjacent discs [19,55]. PMMA has no potential for inducing bone remodeling, osteoinduction, osteoconduction or osteointegration and may block the vascular supply by its presence. Furthermore, cement augmentation may increase the risk of fracture of the vertebra during screw removal [56]. Therefore, both risks and benefits of the cement augmentation should be considered in order to achieve successful outcomes in spinal fusion surgery for osteoporotic patients.

## 10. Expandable Pedicle Screws

Several biomechanical studies demonstrated that expandable pedicle screws improved screw pullout stability compared with standard pedicle screws in osteoporotic spines [57,58]. It has also been reported that expandable pedicle screws can decrease the risk of screw loosening and achieve better fixation strength and clinical results in osteoporotic lumbar spine fusion [59].

However, expandable pedicle screws carry a risk of vertebral bone destruction during screw removal, which may cause nerve root or dural injury [59]. Nevertheless, an application of expandable pedicle screws might be considered in addition to the standard pedicle screws and the cement-augmented screws in surgical treatment for an osteoporotic spine.

## 11. Sublaminar Band

Previous studies have suggested that a sublaminar band can be useful for augmenting screw fixation [11,12]. A biomechanical study using a human thoracolumbar spine revealed that pedicle screws augmented by a sublaminar band provided firmer fixation of screws and a stiffer pedicle screw/rod construct than the same construct without augmentation [12]. Another study showed that a sublaminar band and titanium clamp were useful for reducing and maintaining correction of the thoracic curve in surgical treatment for adolescent idiopathic scoliosis [60].

The polyester sublaminar band is soft and flexible, and the anterior–posterior spinal canal space occupied by the band is less than that taken by a sublaminar wire steel cable, thus avoiding direct spinal cord trauma during sublaminar passage. The flat configuration of the cable distributes the load over a larger contact area under the lamina than metal wires without producing imaging artefacts on postoperative magnetic resonance imaging (MRI) [61]. In contrast to sublaminar wiring, the use of the polyester belt may help reduce the risk of long-term complications associated with bone destruction [11]. Therefore, the sublaminar band may be considered as an alternative augmentation technique for screw fixation in osteoporotic spines [11].

## 12. Hook

Previous biomechanical studies demonstrated that the augmentation of the pedicle screw with a laminar hook can improve stability of the spinal instrumentation [8,9,10]. The combination of pedicle screws and laminar hooks can achieve greater instrumentation stiffness than pedicle screws alone [8,10]. In addition, supplemental offset hooks significantly increase construct stiffness without sacrificing principles of short-segment pedicle instrumentation, and absorb some part of the construct strain, thereby reducing pedicle screw bending moments. Therefore, augmentation with a hook may reduce the risk of postoperative implant failure associated with pedicle screw fixation [9].

Several studies have reported that a central hook–rod construct is effective for spinal osteotomy site closure [62,63]. In addition, the central hook–rod construct may strengthen the overall pedicle screws and rod construct and consequently avoid postoperative screw loosening and implant failure [62,63].

## 13. Discussion

Osteoporosis is a common disease in elderly populations and is a major public health problem worldwide [1]. It is not uncommon for spine surgeons to perform spinal instrumented fusion surgeries for osteoporotic patients [29]. However, in patients with severe osteoporosis, instrumented spine may cause screw loosening, implant failure or nonunion because of bone fragility and decreased pedicle screw stability [4,5]. Previous studies have shown that a decrease in the bone mineral density (BMD) in the spine significantly increases the incidence of screw loosening and nonunion after spinal fusion surgeries [5,64]. Therefore, it is important to consider effective ways to augment screw fixation in patients with osteoporosis in order to achieve optimal postoperative clinical outcomes.

As described above, various screwing techniques to enhance fixation stability have been available for spinal instrumentation surgeries [29]. In addition, many augmentation methods for pedicle screw fixation have been proposed, including those using supplemental hooks [8,10], sublaminar bands [11,12] and other materials, such as PMMA [20]. Biomechanical evidence to support the effectiveness of these augmentation methods has been increasing [8,9,10,11,22,23,24], although clinical evidence is sparse at present.

To achieve success in surgical treatment for osteoporotic spines, meticulous preoperative planning is necessary. It is important to carefully consider the number of pedicle screw placements and the number of fusion levels in order to obtain sufficient spinal stability and rigid internal fixation. It is also essential to preoperatively assess not only the bone quality but also the bone morphology, including fusion/ankylosis of the spine in adjacent levels and spinal deformity and global alignment. The extent of fixation, including the level at which the construct ends, is critical to the prevention of junctional failure. The increased junctional strain at the termination of the construct may lead to accelerated deformity and kyphotic collapse, especially in osteoporotic patients [29,65]. The evaluation of a patient’s daily activity is required to predict the need for postoperative treatment, including the appropriate selection of orthoses and provision of rehabilitation to prevent implant failure, nonunion and adjacent segment problems.

Anterior column support increases load-sharing and decreases strain on the posterior screw fixation construct [29]. Combined anterior–posterior interventions may provide increased stability and consequently reduce the risks of screw loosening and implant failures [29]. Recently, the wide-foot-plate expandable cage system has been developed for reconstruction of the pseudarthrosis after osteoporotic vertebral fracture [66]. This cage has the wide-foot-plate that causes less force on the endplates and hence reduces the risk of cage subsidence [66]. Importantly, this procedure can be performed in a minimally invasive surgical procedure. Therefore, anterior interventions combined with posterior screw fixation can be considered in spinal fusion surgeries in osteoporotic patients.

Drug administration to improve osteoporosis can reportedly enhance pedicle screw stability and spinal fusion after surgery [67,68,69,70]. Several studies have revealed that teriparatide administration to improve the bone quality can reduce pedicle screw loosening and increase the fusion rate following spinal surgery [68,70]. Therefore, perioperative assessments and the proper treatment of osteoporosis may be important for achieving optimal outcomes in spinal instrumentation surgeries in patients with osteoporosis.

## 14. Conclusions

More rigid screw fixation with effective augmentation may reduce the risks of screw loosening and implant failure and provide better postoperative clinical outcomes. Further translational studies should thus be performed to provide firm evidence concerning the optimal surgical treatment of the osteoporotic spine.

## Figures and Tables

**Table 1:** Surgical techniques to reinforce screw fixation in patients with osteoporotic spine.

Reinforcement Strategy	Surgical Technique
Optimal pedicle screw fixation	Appropriate screw diameter and length
	Undertapping
Pelvic fixation	Sacral alar iliac (S2AI) screws
	Intra-sacral buttress screws
Modified screw trajectory	Cortical bone trajectory (CBT) screws
	Penetrating endplate screws
	Groove entry technique
	Hooking screw technique
Placement of substances into the screw hole	Hydroxyapatite stick and granules
	Bone cement (e.g., PMMA)
Modification of screw shape	Expandable pedicle screws
Hybrid posterior constructs	Sublaminar bands with pedicle screws Laminar hooks with pedicle screws
